# Hypertensive Cardiotoxicity in Cancer Treatment—Systematic Analysis of Adjunct, Conventional Chemotherapy, and Novel Therapies—Epidemiology, Incidence, and Pathophysiology

**DOI:** 10.3390/jcm9103346

**Published:** 2020-10-18

**Authors:** Robin Chung, Sara Tyebally, Daniel Chen, Vikas Kapil, J. Malcolm Walker, Daniel Addison, Roohi Ismail-Khan, Avirup Guha, Arjun K Ghosh

**Affiliations:** 1 Cardio-Oncology Service, Barts Heart Centre, St Bartholomew’s Hospital, London EC1A 7BE, UK; robin.chung@nhs.net (R.C.); s.tyebally@nhs.net (S.T.); daniel.chen1@nhs.net (D.C.); 2 Cardio-Oncology Service, University College London Hospital, London WC1E 6HX, UK; malcolm.walker@ucl.ac.uk; 3 Hatter Cardiovascular Institute, University College London, London WC1E 6HX, UK; 4 Barts Blood Pressure Centre of Excellence, Barts Heart Centre, St Bartholomew’s Hospital, London EC1A 7BE, UK; v.kapil@qmul.ac.uk; 5 Centre for Cardiovascular Medicine and Devices, NIHR Barts Biomedical Research Centre, William Harvey Research Institute, Queen Mary University of London, London EC1M 6BQ, UK; 6 Cardio-Oncology Program, Division of Cardiology, The Ohio State University Medical Center, Columbus, OH 43210, USA; daniel.addison@osu.edu (D.A.); avirup.guha@osu.edu (A.G.); 7 Cardio-oncology Program, H. Lee Moffitt Cancer Center, Tampa, FL 33559, USA; Roohi.Ismail-Khan@moffitt.org; 8 Harrington Heart and Vascular Institute, Case Western Reserve University, Cleveland, OH 44106, USA

**Keywords:** cancer, cardiotoxicity, hypertension, cardio-oncology, tyrosine kinase inhibitors

## Abstract

Cardiotoxicity is the umbrella term for cardiovascular side effects of cancer therapies. The most widely recognized phenotype is left ventricular dysfunction, but cardiotoxicity can manifest as arrhythmogenic, vascular, myocarditic and hypertensive toxicities. Hypertension has long been regarded as one of the most prevalent and modifiable cardiovascular risk factors in the general population, but its relevance during the cancer treatment journey may be underestimated. Hypertensive cardiotoxicity occurs de novo in a substantial proportion of treated cancer patients. The pathology is incompletely characterized—natriuresis and renin angiotensin system interactions play a role particularly in conventional treatments, but in novel therapies endothelial dysfunction and the interaction between the cancer and cardiac kinome are implicated. There exists a treatment paradox in that a significant hypertensive response not only mandates anti-hypertensive treatment, but in fact, in certain cancer treatment scenarios, hypertension is a predictor of cancer treatment efficacy and response. In this comprehensive review of over 80,000 patients, we explored the epidemiology, incidence, and mechanistic pathophysiology of hypertensive cardiotoxicity in adjunct, conventional chemotherapy, and novel cancer treatments. Conventional chemotherapy, adjunct treatments, and novel targeted therapies collectively caused new onset hypertension in 33–68% of treated patients. The incidence of hypertensive cardiotoxicity across twenty common novel therapies for any grade hypertension ranged from 4% (imatinib) to 68% (lenvatinib), and high grade 3 or 4 hypertension in <1% (imatinib) to 42% (lenvatinib). The weighted average effect was all-grade hypertension in 24% and grade 3 or 4 hypertension in 8%.

## 1. Introduction

Cancer and cardiovascular disease are the leading causes of death in the developed world, with cancer affecting between 30 to 40% of people in their lifetime [1,2]. However, cancer survival continues to improve due to earlier detection, advanced treatment and improved after-care. Ten-year survival exceeds 50% in the ten most common cancers. Overall, these outcomes translate into 10 million cancer survivors in the USA, 12 million in Europe, including 2.5 million in the United Kingdom [3]. Thus, we move from an encouraging trend among cancer survivors to an appreciation that cancer survivorship at a population level may require life-long intervention to mitigate acquired cardiovascular risks. 

Cardiotoxicity is the umbrella term for a broad range of acute and chronic adverse cardiovascular effects. Cardiotoxicity per se from digitalis, mercurial diuretics and local anesthetics was first reported 70 years ago [4,5,6], but attributed to cancer therapeutics in the 1970s [7]. The most commonly recognised phenotype is left ventricular systolic dysfunction (LVSD), but cardiotoxicity may also manifest as systolic or diastolic left ventricular dysfunction (LVD), propensity to arrhythmia, vascular dysfunction, myocarditis, hypertension or pericardial presentations [8,9,10,11,12].

Hypertension is the most common manifestation of cardiovascular disease with an estimated global burden in the adult population of 26% [13]. Although detailed data about pre-treatment blood pressure (BP) assessment in cancer registries are scarce, a large registry that included 17,712 patients indicated that hypertension was the most frequent comorbidity, with a prevalence of 38% [14]. Treatment of hypertension has powerful favorable effects on major adverse cardiovascular events (MACE), such as coronary heart disease, heart failure and stroke, end-stage renal failure as well as overall mortality [15,16]. More recently there has been a trend in international hypertension guidelines to target lower BP values, largely driven by the recent Systolic Blood Pressure Intervention Trial (SPRINT) study, in which intensive blood pressure control to a target < 120 mmHg in non-diabetic individuals reduced all-cause mortality when compared to a standard target < 140 mmHg (hazard ratio (HR) = 0.73, *p* = 0.003) [17]. There has been a favorable decrease in the prevalence of hypertension and better control of BP in the general population [18], and it is likely that cancer survivors would benefit from management of both hypertension and hypertensive cardiotoxicities.

## 2. Methods

**Figure A1:**
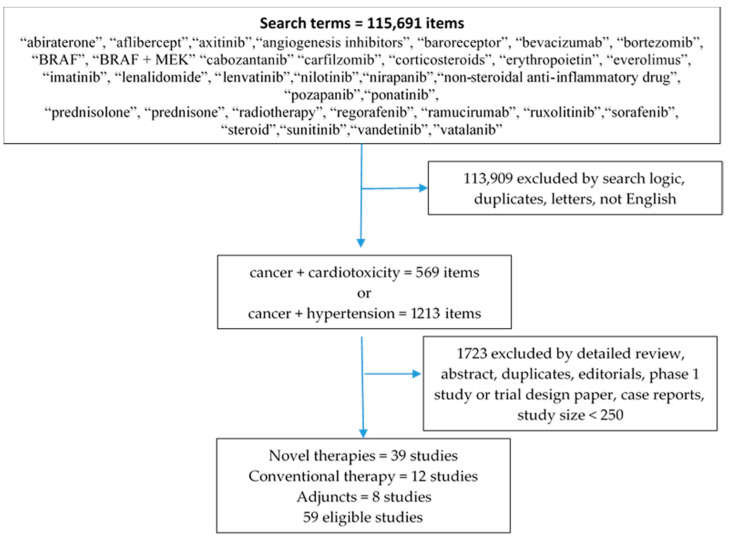
Search terms for conventional, adjunct and novel cancer therapies: Twenty novel (biological and tyrosine kinase inhibitor) cancer treatments known to cause > 5% new incidence of all grade hypertension, and adjunct treatments were selected by consensus; imatinib was included for historical comparison as the earliest approved oral tyrosine kinase inhibitor. The range of cancer therapies were searched against the terms “hypertension”, “cardiotoxicity”, and “cancer”. Inclusion criteria were articles published from 1990 to 2020 in English. Randomized control trials (RCT) including landmark phase 2b/3 studies, observational clinical studies, such as cohort, case-control and cross-sectional studies, as well as meta-analyses and systematic reviews including at least 250 treated patients were included.

We conducted a literature review on the oncological treatments listed in Figure A1. We included the databases on PubMed and MEDLINE using search terms for a range of conventional, adjunct and novel cancer therapeutics. Twenty novel (biological and tyrosine kinase inhibitor) cancer treatments known to cause > 5% new incidence of all grade hypertension and adjunct treatments were selected by consensus; imatinib and rituximab were included for historical comparison as the earliest approved novel tyrosine kinase and monoclonal antibody therapies. The range of cancer therapies were searched against the terms “hypertension”, “cardiotoxicity”, and “cancer”. Inclusion criteria were articles published from 1990 to 2020 in English. Randomized control trials (RCT) including landmark phase 2b/3 studies, observational clinical studies, such as cohort, case-control and cross-sectional studies, as well as meta-analyses and systematic reviews including at least 250 treated patients were included. Reviews and editorials were included when deemed relevant and related to the topic. Our systematic review was partially based on the Preferred Reporting Items for Systematic Review and Meta Analysis (PRISMA) method, but limited to review of hypertension in cancer. Hypertension is an adverse treatment effect, but as it is neither a primary nor secondary outcome measure per se in cancer trials, full PRISMA meta-analysis checklist items such as risk of bias, summary measures (e.g., risk ratio), heterogeneity (I^2^*)* measures, or forest plot were not extracted in our analysis.

### Definition

Various international standards committees have proposed different definitions for hypertension and treatment thresholds in the general population as well as in cancer populations. Thus there is no standard definition for hypertensive cardiotoxicity. A comparison of European Society of Cardiology (ESC) 2018, American College of Cardiology/American Heart Association: ACC/AHA 2017 and National Cancer Institute (NCI) common terminology criteria for adverse effects (CTCAE) version 5 [19] classifications for hypertension is presented in Table 1.

The ESC 2018 guidelines define grade 1 hypertension as an office blood pressure of systolic blood pressure (SBP) ≥ 140 and/or diastolic blood pressure (DBP) ≥ 90 mmHg. The JNC 8 guidelines define stage 1 hypertension as SBP ≥ 130 mmHg or DBP ≥ 80 mmHg. The ACC/AHA 2017 [22] and ESC 2018 [21] guidelines differ because the ACC/AHA proposes a staging classification based on blood pressure thresholds only, whereas the ESC 2018 guidelines propose risk and stage-based thresholds based on blood pressure thresholds and risk factors for target organ damage, chronic kidney disease or cardiovascular disease. National Cancer Institute (NCI) common terminology criteria for adverse effects 2017 (CTCAE version 5) classification standardizes the grading of adverse effects from grade 1 (mild) to grade 5 (death). The CTCAE grade 1 falls within the mild “pre-hypertensive” phase and does not require treatment. Grade 2 moderate hypertension is defined as a systolic blood pressure 140–159 mmHg or diastolic blood pressure 90–99 mmHg or a symptomatic diastolic increase of 20 mm Hg whereby drug monotherapy may be indicated. Grade 3 severe hypertension is defined as systolic blood pressure greater than 160 mmHg or diastolic blood pressure greater than 100 mmHg requiring hospital admission and two or more drugs. Grade 4 life-threatening hypertensive emergencies require hospital admission for urgent intervention, usually intravenous anti-hypertensives, with invasive arterial pressure monitoring. Grades 3 and 4 are grouped in the literature as “serious adverse events” because affected patients require urgent intervention with escalating drug therapy and high dependency monitoring, respectively. 

## 3. Epidemiology

Both cancer and hypertension become increasingly co-prevalent with age. The overall global burden of hypertension in the general adult population in 2000 was 26.6% (male 26.6%, female 26.1%), with a projected global increase in the adult population by 2025 to 29.2% [13]. Hypertension becomes more common with increasing age—more than 60% of adults aged 60 or older and 75% of those aged more than 70 are hypertensive [21,23,24]. Similarly, cancer becomes more prevalent with increasing age—more than half of all cancers are diagnosed in people older than 65 [24].

The incidence of newly-diagnosed hypertension in cancer patients has been quantified retrospectively. At baseline, Fraeman et al. [25] reported the incidence of new-onset moderate (CTCAE grade 2 systolic blood pressure (SBP) > 150–160 mmHg or diastolic blood pressure (DBP) > 100 = 110 mmHg), severe (CTCAE grade 3 SBP > 160–180 mmHg or DBP > 110–120 mmHg) and crisis level (CTCAE grade 4 SBP > 180 mmHg or DBP > 120 mmHg) hypertension as 29%, 16%, and 4%, respectively, across all cancer types. During treatment (cytotoxic chemotherapy or targeted therapies) across all cancer types, the incidence increased more than three-fold. During treatment, moderate hypertension was documented in 90 cases per 100 person-years, severe hypertension in 40 cases per 100 person-years, and crisis level hypertension in 9 cases per 100 person-years. By cancer type, renal, head and neck, and gastric cancers had the highest incidence of crisis level (19.5, 18.4, and 16.3 cases per 100 person-years, respectively) compared to the soft tissue sarcomas with the lowest rate 4.8 cases per 100 person-years. Although hypertension is a well-established renal cancer risk, Fraeman et al. documented de novo hypertension during cancer treatment. The risk of severe or crisis level (CTCAE grade 3 or 4) hypertension increased with successive treatment escalation, e.g., hazard ratio (HR) = 1.98, 2.99, 3.20, 7.93 and 8.01 for first-line cytotoxic chemotherapy, first-line targeted therapy, first-line combination (cytotoxic + targeted), second-line targeted therapy and third-line targeted therapy regimens, respectively [25].

## 4. Hypertensive Cardiotoxicities of Cancer Therapies

**Figure 1:**
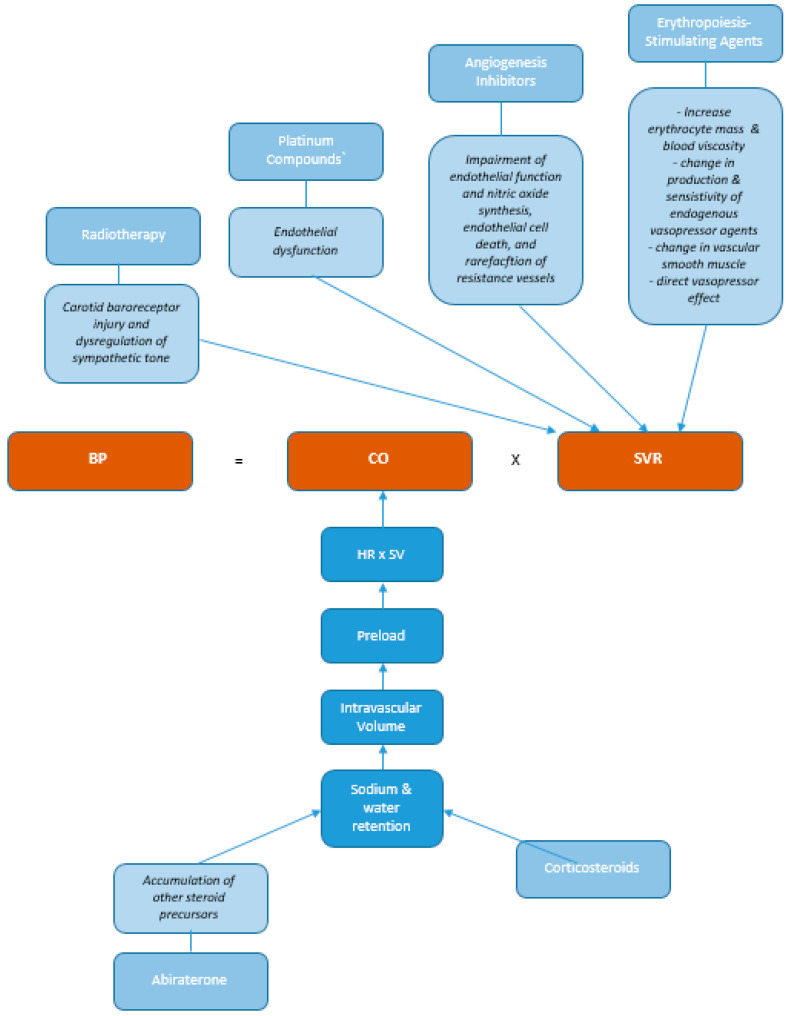
Pathophysiology of cancer therapies leading to hypertension. Outline of the pathophysiology of cancer therapies leading to hypertension. Cancer therapies can have various impacts on the systemic vascular resistance and cardiac output which ultimately has an effect on the blood pressure. BP: Blood pressure, CO: Cardiac output, SVR: Systemic vascular resistance.

As noted above, conventional and emerging novel cancer therapeutics, as well as adjunctive treatments, give rise to hypertension as an important cardiovascular adverse effect by several mechanisms (see Figure 1). Adjunct cancer treatments including glucocorticoids and erythropoiesis stimulating agents (ESA) commonly increase blood pressure [26]. Conventional cancer chemotherapy treatments such as vinca alkaloids, platinum compounds, taxanes, as well as serine-threonine kinase mammalian target-of-rapamycin (mTOR) inhibitors, and head and neck cervical radiotherapy are all recognized hypertension precipitants [27]. More recently, novel targeted cancer therapies, including vascular endothelial growth factor (VEGF) inhibitors, proteasome inhibitors (PI), and tyrosine kinase inhibitors (TKI), have been recognized as significant triggers of hypertension (Table 2) [9]. Importantly, co-existent hypertension is an identified risk factor for other cardiotoxicities such as Human Epidermal Growth Factor receptor 2 (HER2-associated) LVSD cardiotoxicity [28] though it is not known whether treating BP to conventional or more aggressive targets immediately prior to receiving cancer therapeutics reduces the risk of these cardiotoxicities as has been shown in other populations of diabetic, non-diabetic and chronic kidney disease patients [15,16,17]. 

### 4.1. Pathophysiologic Mechanisms

**Figure 2:**
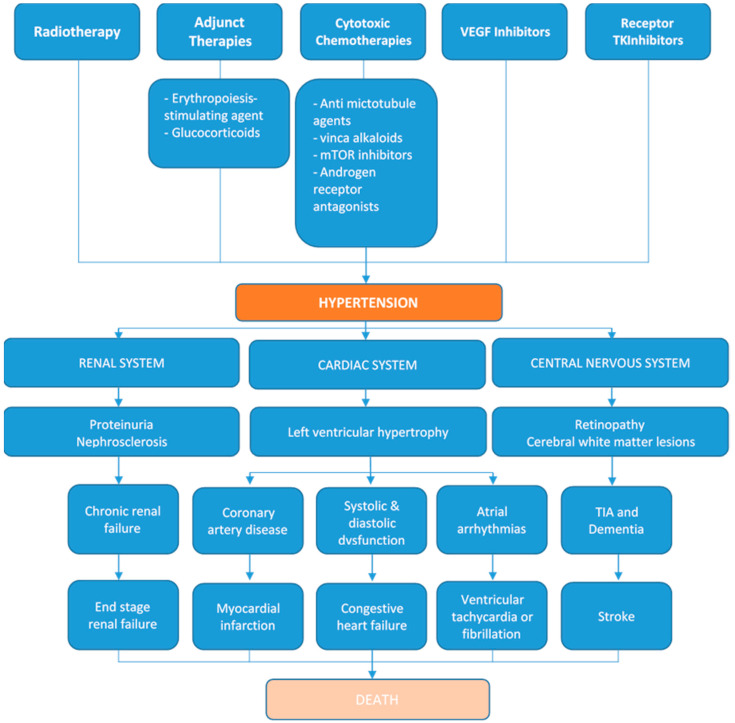
Cancer therapies causing hypertension and their subsequent effects ranging from target-organ damage to final common pathway of end-stage disease [64]. Multiple therapies in cancer have hypertensive effects. This has profound implications on the renal, cardiac and central nervous system. Over time, this can lead to end organ damage and subsequent death. (mTOR: mammalian Target of Rapamycin, TKI: tyrosine kinase inhibitors, VEGF: Vascular Endothelial Growth Factor, TIA: Transient ischemic attack).

The final common pathway for hypertension-mediated target organ damage (HMOD) is a cascade renin angiotensin system (RAS) activation, increased renal vascular resistance and endothelial autoregulatory failure [63]. Hypertensive emergencies are defined as an episode of very high blood pressure values with associated acute HMOD. The resulting organ damage may manifest in malignant hypertension (a hypertensive emergency with severe blood pressure elevation (typically > 200/120 mmHg with grade 3 or 4 hypertensive retinopathy), coronary ischemia, hypertensive heart failure, acute stroke or encephalopathy, acute aortic syndromes, eclampsia or thrombotic microangiopathy syndromes. The final common pathway for hypertension mediated organ damage is illustrated in Figure 2.

### 4.2. Radiotherapy

Radiotherapy to the upper torso confers additional prognostic benefit in head and neck, selected hematological malignancy and breast cancers [65]. Radiotherapy to the abdominal viscera confers both prognostic and disease control in colorectal and gynecological malignancy There are known long term sequelae of head and neck radiotherapy including secondary malignancy, autonomic dysfunction, early cardiac valve fibrosis and accelerated coronary artery atherosclerosis [66,67], whereas abdominal radiotherapy may induce hypertension via renal artery stenosis [68]. Cervical radiotherapy improves survival with chemotherapy in head and neck cancers. However, hypertension manifests as a late effect after cervical radiotherapy due to carotid baroreceptor injury and subsequent dysregulation of sympathetic tone. Radiotherapy for laryngeal or pharyngeal carcinoma attenuated baroreflex sensitivity with a higher mean office blood pressure increased by +24 mmHg) in treated versus control patients (141 mmHg vs. 117 mmHg) without affecting blood pressure variability [69].

### 4.3. Cytotoxic Chemotherapies

The cytotoxic chemotherapies include a broad class of “conventional” anti-cancer agents dating back to the early 1950s and remain in widespread use in up to 30% of cancer regimens even in this current era of targeted therapies [70]. Their anti-neoplastic effects are predicated on non-specific mitotic cell-cycle and inhibition of nuclear (DNA, RNA) replication mechanisms. Drug classes known to induce treatment-associated hypertension include the anti-microtubule agents (paclitaxel, docetaxel, cabazitaxel), alkylating agents (cisplatin, cyclophosphamide and ifosfamide derivatives), vinca alkaloids (vincristine), mammalian target of rapamycin mTOR inhibitors, androgen receptor antagonists (abiraterone) and interferon-alpha. The hypertensive effects of some of these agents are presented in Table 3.

The anti-microtubule agents (paclitaxel docetaxel, cabazitaxel) belong to the taxane class of cancer therapies. They are widely used in solid tumor treatment in breast, prostate, bladder, cervical, Kaposi, gastric, small and non-small cell lung, ovarian, soft tissue sarcoma and germ cell cancers, as well as cancer of unknown primary (CUP). Paclitaxel-associated high grade CTCAE 3 or 4 hypertension was documented in 0.7% of patients [78,79]. Cabazitaxel in metastatic prostate cancer was associated with all-grade hypertension in 4% and grade 3/4 in 2.4% of patients [81].

### 4.4. Alkylating Agents 

The alkylating cytotoxic chemotherapy agents busulfan and bendamustine are known to cause treatment-associated hypertension. Hypertension occurs in 25–36% of patients on busulfan. Bendamustine is associated with a labile blood pressure response resulting in hypertensive emergency in 2.4% (4 of 162), but also hypotension in 3.7% (6 of 162) patients [68]. Cyclophosphamide is implicated in a dose-dependent relationship with fulminant congestive heart failure due to endothelial dysfunction and myopericardial haemorrhage, but it has not been considered an independent risk factor for hypertension in cancer. Symptomatic LVD cardiotoxicity has been reported in up to 25% of patients treated with doses greater than 1.55 g/m^2^/day, compared to less than 3% of patients at lower doses [82]. Cyclophosphamide is not implicated in direct hypertensive cardiotoxicity [68], and in fact may have anti-hypertensive benefit in systemic lupus erthryromatosis [83]. However, as a corollary, ifosfamide nephrotoxicity may explain why adults, and in particular, 10% of children from small long term follow up studies develop hypertension with its use [84]. 

### 4.5. Platinum Compounds

Cisplatin-induced hypertension and acute thrombotic events are due to endothelial dysfunction and thromboxane-A2 production. Platinum compounds are detectable more than 10 years after treatment and this may account for the unpredictable long term risk of hypertensive and vascular cardiotoxicity [85]. Sagstuen et al. reported in testicular cancer patients higher rates of hypertension in those who had received cisplatin compared to surgical treatment only (cisplatin < 850 mg = 50%, cisplatin > 850 mg = 53%, surgery 39%). The similar incidence of hypertension in low- and high-dose cisplatin may be partially attributed to its renal toxicity profile in up to one-third of patients [86]. Concordantly more patients in each group were treated with anti-hypertensives (cisplatin < 850 mg = 8.1%, cisplatin > 850 mg = 11.8%, surgery = 7%) with odds ratio cisplatin < 850 mg = 1.62 (95% CI 1.14–2.32) and cisplatin > 850 mg 2.37 (95% CI 1.4 to 4.01) [71].

### 4.6. mTOR and Interferon Alpha

The mammalian (mechanistic) target of rapamycin (mTOR) inhibitors everolimus, sirolimus, and temsirolimus are effective anti-cancer agents in neuroendocrine, breast and renal cell carcinomas. They may be more familiar to cardiologists as locally active anti-proliferative drug eluting coatings on coronary stents which reduce acute stent thrombosis and in stent restenosis. mTOR inhibitors suppress mTORC1 kinase complex, thereby suppressing anabolic protein synthesis and activating catabolic autophagy. In a review article by Bendtsten et al. [87] assessing the incidence of hypertension in patients with metastatic renal cell carcinoma, everolimus can cause hypertension in 2% of patients grade 3/4 vs. 10% all grades. When used in combination with lenvatinib, the incidence of hypertension rose to 42% all grades vs. 13% grade 3/4. 

Sirolimus and its pro-drug temsirolimus are not known to have hypertensive effects. Interferon alpha in metastatic renal cell carcinoma CTCAE 3 or 4 in 1%, all grade in 4–9% [77].

### 4.7. Abiraterone

The androgen inhibitor abiraterone is a novel cancer agent effective in (metastatic) prostate cancer. It selectively inhibits androgen steroid synthesis. Its hypertensive effect arises from accumulation of other steroid precursors and provokes hypertension in 20% of treated patients. The incidence of all-grade and high-grade hypertension by the abiraterone was 23–26.2% and 6.9–9%, respectively; these were significantly increased compared with placebo (RR, 1.79; 95% CI, 1.45–2.21; *p* < 0.001 and RR, 2.19; 95% CI, 1.73–2.78; *p* < 0.001) [33].

### 4.8. Rituximab

Rituximab is a chimeric monoclonal antibody to the B-cell marker CD20 and is the first common biologic agent approved in 1997 for the treatment of B-cell lymphoma and lymphoproliferative disorders. By binding to CD20, rituximab depletes subpopulations of peripheral B cells of which several mechanisms have been postulated, including cell-mediated and complement-dependent cytotoxicity and promotion of apoptosis. It is administered as an intravenous infusion. Hypotension is among the most common side effect occurring as an infusion reaction. This includes cytokine release syndrome (fever, rigors, urticaria, bronchospasm, throat swelling, nausea, fatigue) occurring predictably and with decreasing frequency with repeated dosing (1st cycle 77%, 4th cycle 30%, 5th cycle 14%), with severe reactions in 0.04 to 0.07%. Thus, hypertension with rituximab is uncommon—reported in 5% of cases, with severe cases (CTCAE grade 3–4) in less than 1% of cases [88,89,90].

### 4.9. Vascular Endothelial Growth Factor (VEGF) Inhibitors

Vascular endothelial growth factors (VEGF) and their receptors (VEGFR) play a critical role in promoting pro-mitotic pathways and angiogenesis, endothelial cell survival and vascular permeability. These functions are critical during development and subsequent physiologic homeostasis but can become pathogenic in cancers and several ophthalmic diseases [91]. This subsequently led to the development of VEGF inhibitors, with bevacizumab being the first anti-VEGF monoclonal antibody available for clinical practice initially in metastatic colorectal cancer. Hurwitz et al. [92,93] in their landmark trial, reported an association between bevacizumab and the development of arterial hypertension of any grade in 22.4% and grade 3 or 4 in 11%, including hypertensive emergencies manifest as posterior reversible leukoencephaly [94]. Subsequent meta analyses of bevacizumab in other trials have confirmed similar incidence of all grade hypertension in 25% and grade 3/4 in 8% [36]. 

The induction of hypertension with VEGF inhibitors is considered a mechanism-dependent toxicity and may reflect both on-target and ‘off-target’ effect of these medications and the overlap in the cardiac and cancer kinome [95]. Whereas adjunctive therapy-induced hypertension (and by implication abiraterone) is mediated via sodium retention and increased preload, ESA, radiotherapy, platinum, and novel angiogenesis inhibitor-induced hypertensive cardiotoxicity are mediated via multiple pathways that increase systemic vascular resistance (Figure 2) In some instances, hypertension may represent a marker of anti-cancer efficacy for patients with renal and neuroendocrine cancers [96,97,98].

Several mechanisms have been proposed to explain this effect of improved outcomes associated with induced hypertension. The stimulation of endothelial cells through VEGFR leads to both an augmented transcription of nitric oxide (NO)-synthase gene and the phosphorylation of NO-synthase, resulting in an increased production of NO [99]. NO is a vasodilator, and so decreased NO synthesis promotes vasoconstriction and increased peripheral resistance, thereby increasing blood pressure. In VEGF inhibitor-induced hypertension, NO synthesis is thought to be suppressed. For example, patients diagnosed with renal cell cancer receiving VEGF inhibitors were found to have reduced urinary excretion of NO metabolites [100]. NO is also involved in tubulo-glomerular feedback, pressure natriuresis and sodium balance, hence decreased levels may subsequently lead to the development of hypertension through sodium retention and direct renal effects [101,102].

Capillary rarefaction, defined as a reduced spatial density of microvascular networks, is another possible mechanism. This feature is known to be a common finding in essential hypertension. Patients diagnosed with colon cancer treated with bevacizumab were found to have a reduction in capillary density in the dorsum of the finger after 6 months of therapy [101]. Moreover, increased production of reactive oxygen species with consequent increase in oxidative stress may account for an additional mechanism in VEGF inhibitors-induced hypertension [103,104]. A role for a renin–angiotensin system (RAS) in VEGF inhibitor-induced hypertension was also hypothesized, but most of the evidence available in both human and experimental models showed a counter-regulative suppression of RAS in this setting [105,106].

### 4.10. Tyrosine Kinase Inhibitors (TKI)

The tyrosine kinase inhibitors (TKI) most notorious for causing hypertension are those that target the vascular endothelial growth factor (VEGF) signaling pathway. They also inhibit other growth factors and kinases including c-kit protein, platelet-derived growth factor receptor (PDGFR), and FMS-like tyrosine kinase-3 [107,108]. The B-rapid accelerating fibrosarcoma (BRAF) (dabrafenib, vemurafenib, encorafenib) and mitogen extracellular signal-regulated kinase (MEK) inhibitors (trametinib, cobimetinib, binimetinib) are serine-threonine kinase inhibitors that are active against V600 mutations in melanoma and colorectal carcinomas [39]. Hypertension of all grades remains a significant treatment adverse effect with these drugs, with evidence of a class effect. Across the receptor TKI range, any grade of hypertensive reaction is common, with the severest grade 3 or 4 mandating some form of urgent intervention to manage the hypertensive reaction. 

The likely mechanisms related to TKI-hypertension are largely similar to VEGF inhibitors, given the overlap in cancer therapeutic mechanisms. Endothelin signaling may also play an important role. Normal VEGF signaling mediates endothelial homeostasis, and VEGF inhibition leads to endothelial dysfunction, stimulating the release of Endothelin-1 (ET-1), a potent vasoconstrictor that may play a role in mediating hypertension [109]. Kappers et al. have reported a parallel rise in ET-1 and hypertension in humans during treatment with sunitinib [106]. Evidence to support this mechanism derives from studies using macitentan, an endothelin receptor (ET-1) antagonist, which inhibits the rise in blood pressure induced by sunitinib [110]. Sunitinib treatment was also associated with a fall in plasma renin concentration and plasma renin activity, without changing the plasma concentrations of aldosterone. Thus it is possible that mineralocorticoid-receptor activation may also play a role in the development of sunitinib-induced hypertension [106]. An observational study by Alivon et al. [111] elegantly demonstrates that large artery properties are affected by vascular signaling pathway inhibition by sunitinib or sorafenib. These drugs cause an increase in arterial stiffness and this increase is partially independent of the blood pressure change. Ibrutinib, an irreversible inhibitor of Bruton’s tyrosine kinase, is indicated in advanced B-cell malignancy. It is implicated in new onset hypertension in 49% and CTCAE grade 3 hypertension in 16% [45,46,47,48,49].

Hypertension manifests as a rise in diastolic blood pressure and proteinuria predict adequate dosing and survival [77,96]. Post-treatment hypertension sorafenib-induced hypertension confers longer progression-free survival (PFS) and overall survival (OS) [59]. Similar prognostically beneficial effects have been observed in other TKIs such as axitinib [112]. 

The incidence of new onset hypertension among a representative group of novel monoclonal and tyrosine kinase cancer therapies ranged from 4% (imatinib) to 68% (lenvatinib), and grade 3 or higher in 1% (imatinib) to 42% (lenvatinib) (see Table 2). Nilotinib, bortezomib, and ruxolitinib were associated with all grade hypertension in 6–9% and high grade CTCAE 3 or 4 hypertension in 1–7%, respectively. Carfilzomib, sorafenib and sunitinib were associated with moderate increased incidence of all grade hypertension 12–21% and high grade CTCAE 3 or 4 hypertension in 4–7%, respectively. Vandetanib, cabozantinib, and vatalanib were associated with all grade hypertension of 24–29% and high grade CTCAE 3 or 4 hypertension in 7–22%, respectively. Pazopanib, axitinib, aflibercept, and regorafenib were associated with all grade hypertension in 36–44% and high grade CTCAE 3 or 4 hypertension in 7–17%. Ibrutinib and lenvatinib were associated with the highest rate of all grade hypertension 49–68% and high grade CTCAE 3 or 4 hypertension in 16–42%, respectively. The BRAF and MEK inhibitors, typically used in combination therapy for melanoma with V600 mutations, evoked a hypertensive response of any grade in 20.6% and CTCAE grade 3 or 4 in 10.1% [39]. 

**Figure 3:**
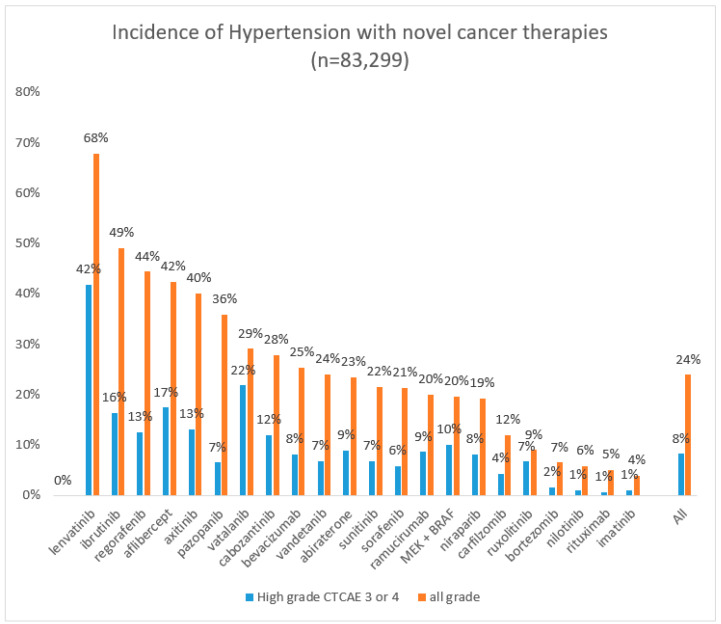
Graph showing incidence of hypertensive cardiotoxicity (high grade versus all grade) with various novel cancer therapies. Incidence is ordered from highest to lowest for all grade hypertension; the bar for all is a weighted average calculation across all novel drug types in our analysis.

We calculated a weighted average across 83,000 patients to illustrate the overall hypertensive class effect of novel cancer therapies. Figure 3 shows the distribution at a glance for individual novel cancer therapies as well as the weighted class effect of all grade hypertension. The overall incidence of all grade hypertension was 24%, and severe hypertensive cardiotoxicity, CTCAE grade 3 or 4, occurred in 8% of patients.

### 4.11. Adjunct Treatments 

The magnitude of the hypertensive effect of adjunct cancer treatments is summarized in Table 4. The joint American Society of Clinical Oncology/American Society of Haematology (ASCO/ASH) guidelines recommend an erythropoiesis stimulating agent (ESA) such as recombinant erythropoietin (EPO) or darbopoeitin (DPO) for chemotherapy-induced anemia on treatment where hemoglobin (Hb) < 10 g/dL. There is a “black box” warning applied to ESA use in cancer patients who are not on treatment due to increased mortality [113]. EPO may cause hypertension in one-third of patients within 16 weeks of treatment due to increased peripheral vascular resistance from direct vasopressor, increased blood viscosity, and reduced vasodilator effects [26].

Endogenous glucocorticoid steroids are derived from cholesterol and synthesized by the adrenal glands to modulate gene transcription in metabolic and immunological functions in multiple cell lines. Synthetic glucocorticoids such as prednisolone/prednisone or methylprednisolone are useful for immunosuppression and anti-emetic effects in rheumatology, transplant and hem-oncology patients. Glucocorticoids may cause multiple adverse effects including poor wound healing, insulin resistance, adrenal suppression, acute psychosis, lipodystrophy, osteoporosis, gastro-intestinal ulcer, and hypertension. The hypertensive effect is attributed to increased sodium retention via stimulation of the mineralocorticoid receptors and increased vascular tone via upregulation of angiotensin-1 receptors. Long term, higher dose (prednisolone equivalent of >15 mg/day for >60 days) glucocorticoid use results in hypertension in a quarter of patients (low dose = 33.9 and high dose = 41.9 cases per 1000 patient-months) [122,123,124]. In a retrospective study conducted by Chari et al. investigating the incidence and risk of hypertension in patients newly treated for multiple myeloma, they found that 54% of patients with multiple myeloma with co-existing diabetes had hypertension compared to 36% in those without diabetes. This is of particular concern given that the routine use of corticosteroids in myeloma therapy can lead to new diagnoses of diabetes. Typical regimens such as bortezomib (Velcade^®^), cyclophosphamide and dexamethasone (VCD) may thus increase the risk of developing diabetes and hypertension in patients treated for myeloma [125]. Of note, dexamethasone has much less mineralocorticoid activity than prednisolone, and therefore has less of an acute BP effect. In patients where dexamethasone is an acceptable alternative to prednisolone in their adjunctive or standard cancer therapeutic regime, this in-class switch may produce less BP elevation. 

## 5. Gaps in Evidence—Late Effects, Reversibility and Recurrence after Treatment

The time course and persistence of hypertensive cardiotoxicity is currently not known after cancer treatment with novel therapies. Whereas cardiotoxic LVD has been (controversially) classified as type I (irreversible) and type II (reversible) to distinguish between anthracycline-mediated and other (e.g., HER2 blockade-mediated forms), there is no equivalent temporal classification for hypertensive cardiotoxicity. Hermann et al. proposed a similar taxonomy for vascular cardiotoxicity according to sustained injury (type I) or transient dysfunction (type II) following adverse arterial thrombotic events [22]. Type I vascular cardiotoxicity is observed in both conventional treatments (cisplatin, bleomycin, vincristine) and novel treatments (nilotinib and ponatinib) causing progressive occlusive arterial disease, whereas type II vascular cardiotoxicity is observed in 5-fluorouracil, capecitabine, everolimus, bevacizumab, and rituximab treatment. Although platinum compound hypertension is attributed to its systemic persistence, it is unknown whether these vascular cardiotoxicities also translate into a sustained or transient hypertensive response in novel treatments. Long term post-treatment clinical follow up and big data linkage studies are required to characterize the time course. This emerging data will inform whether there is a chronic type I (sustained) hypertensive response or an acute type II (transient) hypertensive episode following targeted or small molecule cancer therapies.

## 6. Discussion 

Hypertension is a major contributor to the morbidity and mortality of cardiovascular disease. One quarter of the global adult population is hypertensive [13], and the SPRINT trial demonstrated that aggressive blood pressure control to a systolic pressure < 120 mmHg resulted in significantly reduced mortality in a cancer-free population [17]. Conventional and adjunct cancer treatments can provoke hypertension, but until recently this has received little attention given the requisite primary focus on cancer outcomes. State-of-the-art novel cancer therapies based on monoclonal antibody, tyrosine kinase and other molecular targets have dramatically improved survival in advanced cancers, but similarly highlighted the importance of secondary hypertension as a contributor to LV dysfunction as well as a major adverse cardiotoxicity in its own right.

We have shown that significant new onset hypertension occurs across the range of cancer treatment, ranging from adjuncts such as ESA and glucocorticoids, as well as in conventional chemotherapies and novel cancer therapies. Hypertension (by various metrics) occurs in one-third to one-half of cancer patients treated with adjuncts or conventional chemotherapies. In a representative group of 20 novel therapies, hypertension occurred as an overall average ‘class effect’ in 24% and severe grade 3 or 4 hypertension in 8% of patients. The incidence of hypertensive cardiotoxicity for any grade hypertension ranged from 4% (imatinib) to 68% (lenvatinib), and high grade 3 or 4 hypertension in <1% (imatinib) to 42% (lenvatinib). 

The mechanisms are diverse, ranging from natriuresis effects, renin angiotensin system activation, endothelial nitric oxide mechanisms to cardiac and cancer kinome interplay. There is a clear need for further randomized controlled trials to understand whether pre-treatment (to either standard or more aggressive targets) immediately before initiating cancer therapy prevents both hypertension-related cardiotoxicities (such as LVD) and also other arrhythmogenic or inflammatory cardiotoxicities. Furthermore, it is not known whether concomitant treatment of hypertension *in a cancer population* has the same beneficial effect on future CVD that is apparent in the general population. Finally, although new onset hypertension requiring treatment presents an important clinical problem, hypertension per se in certain novel cancer therapy regimens predicts improved progression free and overall survival [126].

This study contributes to our understanding of hypertensive cardiotoxicity by quantifying the effect of old and new cancer treatments across more than 80,000 patients in a single work. On a practical basis, the information presented may be useful in a clinical setting to both oncologists and cardiologists alike as we have quantified the effect based on standard clinical CTCAE criteria and incidence rather than the usual relative risk ratio employed in most systematic reviews.

## 7. Conclusions

Cancer therapy-associated hypertensive cardiotoxicity occurs in a substantial proportion across the range of adjunct, conventional and novel cancer treatments. In a sample of novel cancer therapies, the overall incidence of any grade of hypertension was 24% and high grade 3 or 4 hypertension was 8%. High grade hypertension generally warrants treatment, but also signals a favorable prognostic marker in certain cancers. Future studies should explore the potential benefit of treating hypertensive cardiotoxicity on cardiovascular outcomes.

## Figures and Tables

**Table 1:** Comparison of ACC/AHA [20], ESC 2018 [21] and NCI Common Terminology Criteria for Adverse Events 2017 (CTCAE version 5) [19] classification for hypertension.

Classification					
CTCAE Qualitative description	Asymptomatic or mild symptoms	Minimal or moderate symptoms limiting activities of daily living	Severe or medically significant, may require hospitalization not life threatening	Life threatening or urgent intervention indicated	Death related to adverse effects
CTCAE grade Hypertension	CTCAE grade 1 Adult SBP 120–139 or DBP 80–89	CTCAE grade 2 SBP 140–159 or DBP 90–99 if previously normal. Symptomatic increase DBP 20 mmHg or > 140/90	CTCAE grade 3 SBP ≥ 160 mmHg or DBP ≥ 100 mmHg	CTCAE grade 4 Life-threatening consequences: Malignant hypertension (retinopathy with BP > 200/120), hypertensive crisis, permanent neurologic deficit	CTCAE grade 5 Death
CTCAE Indicated Treatment	None	Drug monotherapy	More than 1 drug, or increase current therapy	urgent intervention	
ACC/AHA	Normal SBP < 120 and DBP < 80	Elevated SBP 120–129 and DBP < 80	Stage 1 SBP 130–139, or DBP 80–89	Stage 2 SBP ≥ 140 or DBP ≥ 90	
ESC 2018 grade	Normal SBP 120–129, and/or DBP 80–84	High normal SBP 130–139 and/or DBP 85–89	Grade 1 SBP 140–159 and/or DBP 90–99	Grade 2 SBP 160–179, and/or DBP 100–109	Grade 3 SBP ≥ 180, and/or DBP ≥ 110

ACC = American College of Cardiology; AHA = American Heart Association; DBP = diastolic blood pressure; ESC = European Society of Cardiology; NCI = National Cancer Institute; SBP = systolic blood pressure.

**Table 2:** Hypertensive cardiotoxicities in novel cancer therapies.

Drug	Number of Patients	All Grades Hypertension %	CTCAE 3–4 Hypertension %
Abiraterone [29,30,31,32,33]	8323	23.4%	8.9%
Aflibercept [34]	4451	42.4%	17.4%
Axitinib [35]	1908	40%	13.1%
Bevacizumab [36]	21,902	25%	8%
Bortezomib [37]	2509	6.5%	1.6%
BRAF + MEK inhibitors [38,39]	791	20.6%	10.1%
Cabozantinib [40]	1514	28%	7%
Carfilzomib [41,42,43]	2594	12%	4.3%
Imatinib [22,44]	280	4%	0.4%
Ibrutinib [45,46,47,48,49]	1364	49.1%	16.3%
Lenvatinib [50]	261	67.8%	42.9%
Nilotinib [44,51,52]	997	5.9%	1.1%
Niraparib [53]	367	19.3%	8.2%
Pozapanib [54]	1651	36%	7%
Ramucirumab [55]	3851	20%	9%
Regorafenib [56]	1069	44%	12.5%
Ruxolitinib [57,58]	220	9.3%	6.7%
Sorafinib [59]	20,494	21%	6%
Sunitinib [60]	4999	22%	7.9%
Vatalanib [61]	422	29%	22%
Vandetanib [62]	3154	24%	6.8%

BRAF = B-Rapid activating fibrosarcoma; CTCAE = Common Terminology Criteria for Adverse Effects; MEK = mitogen extracellular signal-regulated kinase.

**Table 3:** Legacy and conventional chemotherapy and hypertensive cardiotoxicity.

Drug	N	All Grades Hypertension	CTCAE Grade 3 or 4 Hypertension
Cisplatin [71]	500	50–53%	8.1–11.8% on anti-hypertensive medication
Everolimus [72,73,74,75,76]	985	8.6–10%	0.4–2%
Interferon alpha [77]	360	4%	1%
Paclitaxel [78,79,80]	717	0.8%	0.7%

CTCAE = Common Terminology Criteria for Adverse Effects.

**Table 4:** Adjunct therapies and hypertensive cardiotoxicity data from [114,115,116,117,118,119,120,121].

	Incidence of Hypertension	Magnitude	Mechanism	Indication
Erythroypoeisis stimulating agents [26]	33%	SBP + 5 to +8 mmHg	Increased systemic vascular resistance due to direct vasopressor, increased blood viscosity and nitric oxide vasodilator resistance	Anemia of chemotherapy, Hb < 10 g/dL. Black box warning—contra-indicated in non-chemotherapy cancer anemia
Glucocorticoids [122,123,124]	20–30%		Sodium and water retention Upregulation of AT1 receptors	Immunosuppression in combination with CVP or R-CHOP regimens

AT1 = Angiotensin 1; SBP = systolic blood pressure; Hb = haemoglobin; CVP = cyclophosphomide; R-CHOP = Rituximab-Cyclophophomide, Hydroxy-daunorubicin, Oncovin, Prednisolone.

## Abbreviations

ACC	American College of Cardiology
AHA	American Heart Association
ASCO	American Society of Clinical Oncology
ASH	American Society of Hematology
BP	Blood pressure
BRAF	B-Rapid Accelerating Fibrosarcoma gene
CD	Cluster of Differentiation
CVD	Cardiovascular Disease
CTCAE	Common Terminology Criteria for Adverse Events
CVD	Cardiovascular Disease
CUP	Cancer of Unknown Primary
DNA	de-oxyribonucleic acid
ET-1	Endothelian-1
ESA	Erythropoeisis Stimulating Agents
ESC	European Society of Cardiology
HER2	Human Epidural Growth Factor Receptor 2
HMOD	Hypertension-Mediated Organ Damage
LVD	Left Ventricular Dysfunction
LVSD	Left Ventricular Systolic Dysfunction
MACE	Major Adverse Cardiovascular Event
MEK	Mitogen extracellular signal-regulated kinase
mTOR	mammalian Target of Rapamycin
NCI	National Cancer Institute
NO	Nitric Oxide
NSAID	Non-steroidal anti-inflammatory drug
PDGFR	Plate derived growth factor receptor
RAS	Renin Angiotensin System
RNA	ribonucleic acid
SPRINT	Systolic Blood Pressure Intervention Trial
PRISMA	Preferred Reporting Items for Systematic Reviews and Meta-Analysis
TKI	Tyrosine Kinase Inhibitors
VEGF	Vascular Endothelial Growth Factor
